# Complications and Risk Factors of Patients Undergoing Computed Tomography-Guided Core Needle Lung Biopsy: A Single-Center Experience

**DOI:** 10.7759/cureus.16907

**Published:** 2021-08-05

**Authors:** Waseem M Hajjar, Ibrahim M Fetyani, Rayan M Alqarni, Fahad A Alajlan, Fouad F Bahgat, Sultan R Alharbi

**Affiliations:** 1 Surgery, College of Medicine, King Saud University, Riyadh, SAU; 2 General Surgery, King Saud University, Riyadh, SAU; 3 Interventional Radiology, King Saud University, Riyadh, SAU

**Keywords:** ct-guided core needle biopsy, risk factors, complications, pneumothorax, haemorrhage

## Abstract

Objectives

To determine the risk factors and complications of transthoracic computed tomography (CT)-guided core needle lung biopsy.

Methods

This is a retrospective study of 124 patients who underwent CT-guided core lung biopsy in King Khalid University Hospital (KKUH), Riyadh. This retrospective study was conducted between January 2016 and January 2020. Patient data were collected using a standardized data form that was entered into an Excel sheet in accordance with the variables. The Statistical Package for the Social Sciences software (SPSS, version 24.0 [SPSS Inc., Chicago, IL, USA]) was used to compute for the risk of complications after CT-guided core lung biopsy and perform all statistical comparisons, and the results were reported.

Results

The overall complication rate due to CT-guided core needle biopsy was 34.7% (43) (P<0.001) of the total sample. Of the total complications, 69.76% (n = 30) had pneumothorax, 20.94% (n = 9) had hemorrhage, 6.98% (n = 3) had both pneumothorax and hemorrhage, and 2.32% (n = 1) had both air embolism and pneumothorax. Of all patients who developed pneumothorax, 20% (n = 6) required chest tube insertion. Patients with secondary chronic obstructive pulmonary disease (COPD) had a complication rate of 80% among the whole sample. Lung lesions less than 3 cm had a complication rate of 48.8% (P<0.034). The needle size showed a higher rate of complications between 20 and 18 gauge with 47.4% (n = 9) and 32.4% (n = 34), respectively.

Conclusions

We conclude that CT-guided lung biopsy is a well-established low-risk procedure that is less invasive. However, it still carries a risk of complications with some risk factors, such as small lung lesion size and secondary COPD.

## Introduction

In modern medicine, computed tomography (CT) of the lung is commonly used to screen for primary lung cancer or metastatic cancer, leading to the discovery of more patients with lung masses [1-3]. A minimally invasive procedure such as core needle biopsy reduces the need for open surgical lung biopsy. Such procedures help confirm the diagnosis of suspected lung lesions in addition to several screening programs, one of which is the screening of high-risk groups for lung cancer. It has become widely popular across various institutions and is in constant demand for the diagnosis of different lung diseases [4,5].

Nevertheless, a core needle biopsy is associated with several complications, mainly pneumothorax and hemothorax and their corresponding risk factors [6]. Risk factors include lesion size, depth, the presence of primary emphysema, age, sex, location, shape, the presence of atelectasis, fissure in the needle tract, needle entry angle, number of pleural punctures, the experience of the operator, and procedure duration. Many previous studies have reported on the complications and risks of this procedure, but few have shown the correlation of these factors with the diagnostic yield of different needle gauge sizes used [1,2,7].

Complication rates have varied across different studies, ranging from 8% to 69% [1,2]. Pneumothorax is the most common complication following core needle biopsy [8]. Pneumothorax is defined as the presence of air in the pleural space. This has many causes mainly iatrogenic, especially in CT-guided core needle biopsy. Most pneumothoraces are treated conservatively without chest tube placement and are considered minimal. However, some cases require further attention and subsequent placement of a chest tube [6].

A study reported that the incidence of pneumothorax was 15.4% in patients undergoing the procedure and chest tube placement was needed in only 22.8% of all pneumothoraces [1]. The position of the patient, length of the needle, and history of smoking were the most important risk factors for developing pneumothorax [1,4,8-10].

In some studies, pulmonary hemorrhage, including hemoptysis, intraparenchymal hemorrhage, and hematoma were the most common complications encountered, with an incidence rate of 45.4% in one study [9]. The correlation between this complication and several risk factors was analyzed, maximal diameter and needle length were the most significant risk factors [10]. Some controversy remains when comparing core-needle biopsy to fine-needle aspiration as some studies have reported a higher complication rate in core needle biopsy than in fine-needle aspiration [4]. However, other studies have found that both core needle biopsy and fine-needle aspiration have the same complication rate and suggested the use of the former as it has a better diagnostic outcome [11].

Many studies have shown that CT-guided core needle biopsy is a reliable diagnostic procedure with a low incidence of complications, and it is used for thoracentesis and other therapeutic lung procedures [12]. CT-guided lung biopsy is considered the modality of choice because of its reliability and low complication rate. Usually, biopsy specimens are categorized as malignant, benign, or undetermined. Several factors play a role in the integrity and adequacy of specimens; however, a CT-guided core needle biopsy is known to produce a high diagnostic yield [13-15].

## Materials and methods

Patients

The present study was approved by the institutional review board (IRB) committee of King Saud University. The study was conducted in accordance with international ethical standards. Patient names and other confidential information were not used in this study; therefore, patient consent was waived.

The study was carried out in Riyadh, Saudi Arabia, as a single-center experience in a tertiary university hospital. Data from all patients collected from the Interventional Radiology Department, including that from all patients who underwent CT-guided core lung biopsy from January 2016 to January 2020, were included in this study.

CT-guided core needle lung biopsy technique

**Figure 1:**
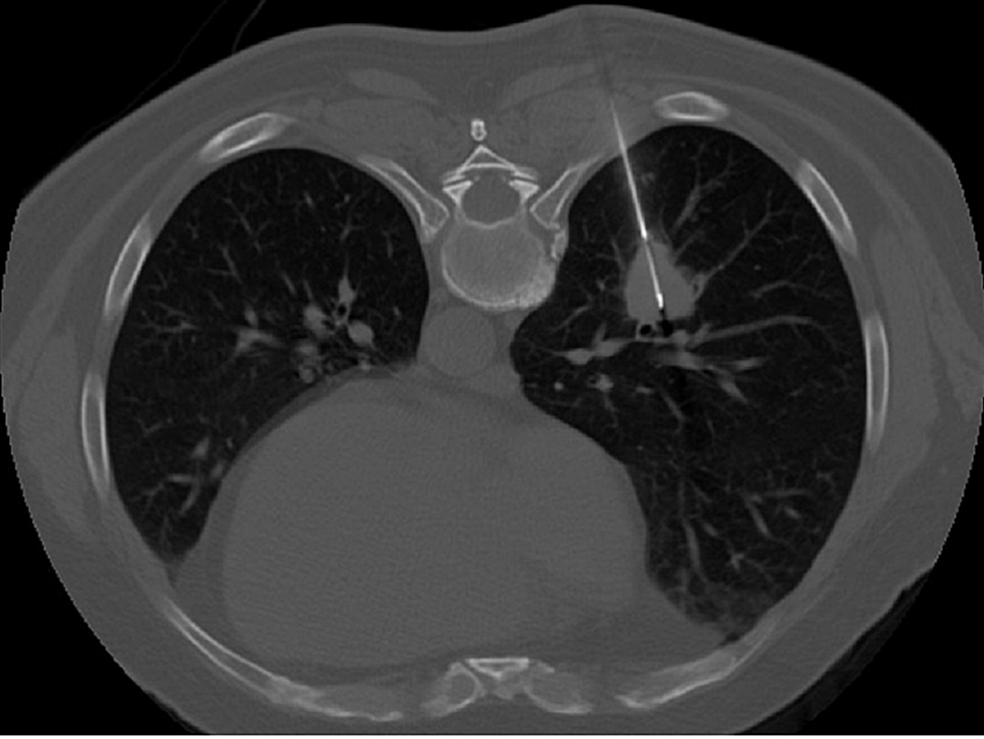
CT-guided lung biopsy of a right lung lesion (prone position). CT - computed tomography

All patients underwent CT-guided needle core lung biopsy by a consultant interventional radiologist. After reviewing previous imaging and obtaining informed consent, the patient was placed in the most suitable position (prone, lateral, or supine) on the CT scan table. Under sterile conditions, local anesthesia, and conscious sedation, lung core biopsies were performed using an 18G or 20G coaxial quick core biopsy needle set (Cook, Bloomington, IN, USA) (Figure 1). The needle set size was determined based on the size of the lesion, background lung disease, performance preference, and item availability. Generally, a 20G needle is used for smaller lesions, chronic obstructive pulmonary disease (COPD) patients, and difficult mass locations to reduce the risk of complications. Post-biopsy CT chest scanning was performed at the end of the procedure to determine if there was a complication. Subsequently, the patient was moved to the surgical ward for observation for 3-4 h. Before discharging the patient, a chest x-ray was also performed to exclude late complications, mainly pneumothorax.

Data collection

This study was conducted as a retrospective study that included 124 patients who underwent CT-guided core lung biopsy. Patient data were collected using a standardized data form. The data were entered into an Excel sheet, and missing data were considered not present. All CT-guided needle biopsies conducted during the period mentioned were reviewed. The outcomes and complications of all the procedures were documented and analyzed.

Inclusion criteria included all patients who underwent CT-guided core needle lung biopsy using an 18G needle or 20G needle in the period mentioned. The exclusion criteria included insufficient data, repetitive data, and canceled procedures. The initial sample size was 301 patients, but this was reduced to 124 patients.

The variables included patients’ demographic data, characteristics, lesion variables, and procedure-related variables. Data were obtained from electronic patient files (e-SiHi) and the Picture Archiving and Communication System (PACS). All CT-guided core needle biopsies conducted during the study period were collected and analyzed, and all data were organized in an Excel sheet and subsequently analyzed using Statistical Package for the Social Sciences (SPSS), and the outcomes were reported. All the mentioned variables and outcomes included were obtained from similar studies that were studied and mentioned in the literature [1-3].

Data and statistical analysis

The risk of complications after CT-guided core needle biopsy and all statistical comparisons were studied using. Statistical analysis was performed using the SPSS version 24.0 software (SPSS Inc., Chicago, IL, USA). Descriptive statistics were reported as mean ± standard deviation for continuous variables and counted as a percentage for categorical values. A total of 124 patients were categorized into two groups based on which needle gauge was used (18G vs. 20G), and then divided into two subgroups depending on the presence of complications. The variables were compared using the chi-square test, Fisher’s exact test, binomial test, and Mann-Whitney U test. The Student’s t-test was used for two independent groups. Statistical significance was set at P < 0.05.

## Results

**Figure 2:**
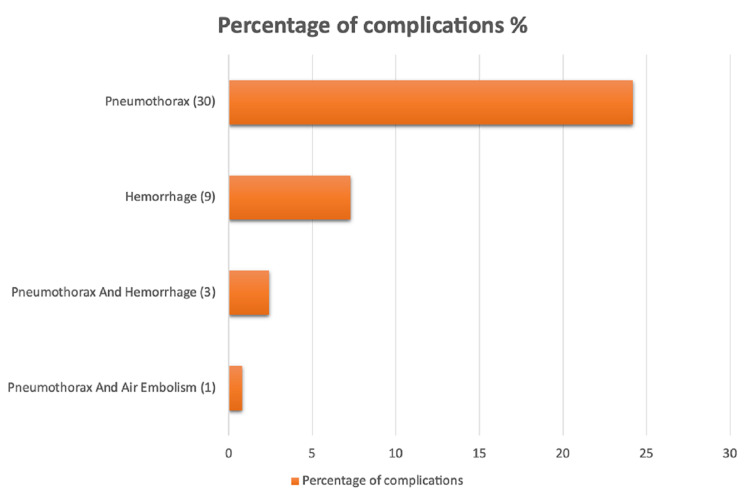
Total percentages of complications related to core needle biopsies.

This study correlated with different variables regarding the presence of complications. The number of cases that developed procedure-related complications had a prevalence of 34.7% (n = 43), with a significant P-value of 0.001. Demographic characteristics, including age, sex, and smoking, showed no significant correlation with complications. Disease-related risk factors namely location and presence of primary emphysema showed no correlation as well. Meanwhile, lung lesion size showed a significantly high incidence of complications in those with less than 3 cm with a p-value of 0.034. Procedure-related risk factors, including patient position, lesion depth, and the number of pleural punctures, showed no statistical significance with respect to the incidence of complications (Table 1). Of the total sample, the incidence of complications and pneumothorax was 24.2% (n = 30), where 7.3% (n = 9) had hemorrhage, 0.8% (n = 1) had both air embolism and pneumothorax, and 2.4% (n = 3) had both pneumothorax and hemorrhage. The total prevalence of different complications demonstrated a significant P-value of <0.0001 (Figure 2). The complications were then correlated with the needle gauge used. A significant difference was found for hemorrhage, which occurred in 5.7% (n = 6) and 15.8% (n = 3) when 18G and 20G were used, respectively, with a P-value of 0.141. Pneumothorax occurred in 23.8% (n = 25) of patients when 18G was used, compared to 26.3% (n = 5) when 20G was used. Additionally, pneumothorax associated with hemorrhage occurred simultaneously in 1.9% (n = 2) of patients when 18G was used and 5.3% (n = 1) when 20G was used. Meanwhile, no complications were seen in 67.6% (71) and 52.6% (10) of patients when 18G and 20G needles were used, respectively (Table 2). There was a single case of an air embolism associated with pneumothorax when an 18G needle was used.

**Table 1:** Patient's biographic and lesion variables.

Variables		Complication		No complication		P-value
		N	%	N	%	
Number of cases		43	(34.7 %)	81	(65.3 %)	0.001
Age	≥60 y	20	(46.5 %)	47	(58 %)	0.221
	<60 y	23	(53.5 %)	34	(42.0 %)	
Gender	Male	32	(74.4 %)	48	(59.3 %)	0.093
	Female	11	(25.6 %)	33	(40.7 %)	
Patient position	Lateral	13	(30.2 %)	11	(13.6 %)	0.064
	Prone	17	(39.5 %)	34	(42.0 %)	
	Supine	13	(30.2 %)	36	(44.4 %)	
Smoking Hx.	Yes	22	(51.2 %)	29	(35.8 %)	0.098
	No	21	(48.8 %)	52	(64.2 %)	
Lung lesion size (in cm)	≤ 3.0	21	(48.8 %)	24	(29.6 %)	0.034
	≥3.1	22	(51.2 %)	57	(70.4 %)	
Lesion depth (in cm)	0	13	(30.2 %)	35	(43.2 %)	0.333
	0.2 – 3	25	(58.1 %)	40	(49.4 %)	
	>3	5	(11.6 %)	6	(7.4 %)	
Comorbidities	None	27	(62.8 %)	53	(65.4 %)	0.863
	Lung disease	10	(23.3 %)	14	(17.3 %)	
	Infections	3	(7.0 %)	7	(8.6 %)	
	Miscellaneous	3	(7.0 %)	7	(8.6 %)	
Severity of the primary emphysema	None	40	(93.0 %)	76	(93.8 %)	0.400
	Moderate	2	(4.7 %)	1	(1.2 %)	
	Severe	1	(2.3 %)	4	(4.9 %)	
Number of pleural punctures	Single	34	(79.1 %)	68	(84.0 %)	0.322
	Multiple	9	(20.9 %)	13	(16.0 %)	
Lesion location	Right lung lesion	22	(51.2 %)	41	(50.8 %)	0.050
	Left lung lesion	20	(46.5 %)	27	(37.9 %)	
	Mediastinal	1	(2.3 %)	13	(11.3 %)	

**Table 2:** Complications in correlation to needle size.

Complications	Size of needle				P-value
	18 (n = 105)		20 (n = 19)		
	N	%	N	%	
Haemorrhage	6	(5.7 %)	3	(15.8 %)	0.141
Pneumothorax	25	(23.8 %)	5	(26.3 %)	0.508
No complication	71	(67.6 %)	10	(52.6 %)	0.207
Pneumothorax and Haemorrhage	2	(1.9 %)	1	(5.3 %)	0.396
Pneumothorax and Air embolism	1	(1.0 %)	0	(0 %)	0.847

## Discussion

CT-guided core needle biopsy has become a widely used tool and diagnostic method for the evaluation of pulmonary lesions. Pneumothorax and pulmonary hemorrhage are by far the most frequent complications. Other rare adverse events include hemorrhage, infection, needle tract seeding, and systemic air embolism [5]. The complication rates in this retrospective study were as follows: the total incidence of complications was 34.7% (n = 43), pneumothorax had the highest incidence among all complications with 69.76% (n = 30) of all patients that developed complications, while pneumothorax developed in only 20% (n = 6), with a P-value of 0.001 with subsequent chest tube insertion. Additionally, hemorrhage occurred in 20.94% (n = 9), pneumothorax associated with hemorrhage in 6.98% (n = 3), and of those, only one patient needed a chest tube insertion, while air embolism associated with pneumothorax was found in only 2.32% (n = 1). We found all complication rates to be consistent with those found in previously published papers [1,4].

Of all risk factors that were studied, smaller lung lesion size and COPD patients had a higher incidence of complications. This correlation with smaller lesions could be explained by repeated attempts to acquire an adequate biopsy sample from the lesion, resulting in more damage to the lung parenchyma and increasing the chance of complications. Regarding the presence of complications in correlation to comorbidities, the incidence of complications in patients with comorbidities compared to healthy subjects was higher, with a prevalence of 37.2% and 34.6%, respectively. Comorbidities were classified into multiple diagnoses, of which only secondary COPD was statistically significant, with a complication incidence of 80% of total secondary COPD patients. Patients with secondary COPD tend to have parenchymal disruption of varying degrees, which could explain the higher incidence of complications in these patients. Other correlated demographic lesion-related and procedure-related factors such as age, smoking, lesion depth, lesion location, and patient position did not show a clear association in our study. However, mixed results are found in the current literature; some studies did not show a correlation with any lesion characteristics, but these are the minority [11].

A variety of studies have compared core needle biopsy and fine-needle aspiration in terms of complications. One systematic review recommends the use of core needle biopsy over fine-needle aspiration because of the same rate of complications with a better diagnostic yield [11]. Additionally, one meta-analysis showed that core needle biopsy did not have significant risk factors for overall complications [4]. However, physicians tend to use a large needle size to obtain large histological samples, as well as to avoid the complications arising from smaller needle sizes, which led to the rarity of using 20G needle biopsies in our institution. Although the correlation between needle size and incidence of complications was not statistically significant in our study, there was a tendency towards a higher complication rate when using the 20 G needle by 47.37% (n = 9). Nevertheless, 18G needles were associated with an incidence of complications of 32.38% (n = 34), which may be due to the use of multiple attempts of needle insertion using the smaller gauge, and the sample size of the complications in the 18G group was significantly higher than that in the 20G group. Thus, this correlation needs more research to obtain a clearer approach.

The limitations of this research include a small sample size and a single-institution study, thus making several risk factors statistically insignificant. The inability to separate multiple co-occurring risk factors correlated individually with complication incidence.

## Conclusions

We conclude that CT-guided lung biopsy is a well-established and low-risk procedure for the diagnosis of lung pathologies. It is also less invasive but associated with a risk of developing complications mainly pneumothorax and hemorrhage. However, small lung lesion size and secondary COPD, both carry a risk of developing complications related to the core needle biopsy, which could be explained by the repetitive attempts to obtain the sample from this small lesion, and also due to the parenchymal pathology in COPD patients.

We recommend further investigations in a multicenter study with a larger sample size to explore the different variables that were not statistically significant but trending toward significance.
